# Reasons for Non-Completion of Health Related Quality of Life Evaluations in Pediatric Acute Myeloid Leukemia: A Report from the Children’s Oncology Group

**DOI:** 10.1371/journal.pone.0074549

**Published:** 2013-09-06

**Authors:** Donna L. Johnston, Rajaram Nagarajan, Mae Caparas, Fiona Schulte, Patricia Cullen, Richard Aplenc, Lillian Sung

**Affiliations:** 1 Division of Hematology/Oncology, Children’s Hospital of Eastern Ontario, Ottawa, Ontario, Canada; 2 Division of Oncology, Cincinnati Children’s Hospital Medical Center, Cincinnati, Ohio, United States of America; 3 Children’s Oncology Group, Monrovia, California, United States of America; 4 Departments of Oncology and Paediatrics, University of Calgary, Alberta Children's Hospital Research Institute, and Haematology, Oncology and Transplant Program, Alberta Children's, Calgary, Alberta, Canada; 5 Loretto Heights School of Nursing, Regis University, Denver, Colorado, United States of America; 6 Pediatric Oncology/Stem Cell Transplant, Children's Hospital of Philadelphia, Department of Pediatrics, University of Pennsylvania, Philadelphia, Pennsylvania, United States of America; 7 Division of Haematology/Oncology, The Hospital for Sick Children, Toronto, Ontario, Canada; Emory University/Georgia Insititute of Technology, United States of America

## Abstract

**Background:**

Health related quality of life (HRQL) assessments during therapy for pediatric cancer are important. The objective of this study was to describe reasons for failure to provide HRQL assessments during a pediatric acute myeloid leukemia (AML) clinical trial.

**Methods:**

We focused on HRQL assessments embedded in a multicenter pediatric AML clinical trial. The PedsQL 4.0 Generic Core Scales, PedsQL 3.0 Acute Cancer Module, PedsQL Multidimensional Fatigue Scale, and Pediatric Inventory for Parents were obtained from parent/guardian respondents at a maximum of six time points. Children provided self-report optionally. A central study coordinator contacted sites with delinquent HRQL data. Reasons for failure to submit the HRQL assessments were evaluated by three pediatric oncologists and themes were generated using thematic analysis.

**Results:**

There were 906 completed and 1091 potential assessments included in this analysis (83%). The median age of included children was 12.9 years (range 2.0 to 18.9). The five themes for non-completion were: patient too ill; passive or active refusal by respondent; developmental delay; logistical challenges; and poor knowledge of study processes from both the respondent and institutional perspective.

**Conclusions:**

We identified reasons for non-completion of HRQL assessments during active therapy. This information will facilitate recommendations to improve study processes and future HRQL study designs to maximize response rates.

## Introduction

Health related quality of life (HRQL) assessments during therapy for pediatric cancer are important. This information is needed to better understand the patient experience and to identify if, when, and how interventions should be considered to improve HRQL [1], [2]. When HRQL is assessed in the context of different treatments, this information may also be used to understand how the regimens differ from the patient and family perspectives and may help with choosing a treatment strategy [2]–[4].

In pediatric cancer, most evaluations of HRQL have been conducted in single center, stand-alone studies. It is possible to embed HRQL evaluations into multi-center co-operative group trials. The advantages of embedding HRQL assessments into a therapeutic trial include efficiency in trial conduct and data management [3]. Furthermore, this approach has the potential for observing interactions among symptoms, calculating quality adjusted survival and cost-effectiveness, and generating new hypotheses [3].

HRQL assessed during intensive treatment is likely to provide informative data. Such intensive treatments may include chemotherapy for acute myeloid leukemia (AML), treatment for relapsed disease, and during hematopoietic stem cell transplantation (HSCT). AAML1031 is a Phase 3 randomized trial being conducted by the Children’s Oncology Group (COG) that involves both chemotherapy and HSCT for patients. An ancillary HRQL aim was embedded in the trial for the purpose of describing HRQL associated with chemotherapy versus HSCT in this intensively treated patient population.

In pediatric oncology, response rates when eliciting information about the symptom experience and HRQL can vary significantly with response rates ranging from 58–98% in recent studies [5]–[10]. Although response rates vary considerably by study, to date, there are no pediatric studies that have described reasons for non-completion of HRQL assessments. With our experience with AAML1031, we had a unique opportunity to describe the reasons behind non-completion of HRQL assessments. The objective was to describe reasons for failure to provide HRQL assessments during an ongoing pediatric AML clinical trial.

## Methods

### Details of AAML1031 HRQL Assessments

AAML1031 (NCT 01371981) is a Phase 3 COG multi-center trial that randomizes patients to receive or not receive bortezomib for *de novo* AML and determines the safety of sorafenib in patients with high allelic ratio FLT3+/ITD. This study uses a 4-course chemotherapy backbone (Induction I, Induction II, Intensification I, and Intensification II). Patients with low risk disease receive 4 courses of chemotherapy while those with high risk disease receive 3 courses of chemotherapy followed by allogeneic HSCT if an appropriate donor is identified. Thus, all patients receive three courses of chemotherapy and then either Intensification II or HSCT.

This study was approved by the research ethics board at all participating institution (table S1). Participants provided written informed consent to participate in this study. Written informed consent was obtained from parents or guardians on behalf of the minor/child participants in this study.

AAML1031 has an embedded secondary aim to assess HRQL of children and adolescents treated with chemotherapy and HSCT for AML. The study also seeks to describe parental stress for these patients. Consent for the HRQL aim is obtained at the same time as consent for the therapeutic study; eligible patients/families who consent to AAML1031 are offered the ability to participate in the HRQL aim as well. The instruments for HRQL assessment are the PedsQL 4.0 Generic Core Scales [11], [12], PedsQL 3.0 Acute Cancer Module [13], and PedsQL Multidimensional Fatigue Scale [13], [14], which measure generic HRQL, cancer-specific HRQL and fatigue respectively. The Pediatric Inventory for Parents is also included and measures parental stress [15], [16]. The estimated time to complete all assessments is 23–32 minutes. There are eight assessment time points stipulated by the protocol as follows: 1) within 14 days of Induction I initiation; 2) ≥ day 21 of Induction II, but prior to start of Intensification I; 3) ≥ day 21 of Intensification I, but prior to start of Intensification II; 4) one month (±7 days) from start of intensification II or HSCT; 5) four months (±1 month) from start of Intensification II or HSCT; 6) 12 months (±1 month) from date of diagnosis; 7) 24 months (±3 months) from date of diagnosis; and 8) 36 months (±3 months) from date of diagnosis.

For the HRQL aim, eligible patients are between 2 and 18 years of age with a parent or guardian who can read in English. Parents or guardians provide proxy assessments for all patients, while for patients ≥5 years of age who can understand English, self-report is also completed if the child provides consent or assent. For all consenting participants, the age-appropriate questionnaires are downloaded from the COG website by institutional clinical research associates (CRAs). Completed forms are reviewed by the CRA for completeness. Responses are entered into the COG database via remote data entry and the original forms are retained by the institutions. Respondents that do not complete a questionnaire (parent or child form) at a particular time point continue to participate in subsequent time points as long as consent to participate is not withdrawn. Respondents continue to submit HRQL assessments until: they complete all questionnaire time points; consent to participate in the HRQL study is withdrawn; or the patient is removed from AAML1031 protocol therapy (for reasons such as relapse, refractory disease or death) in which case they are no longer eligible for the HRQL study.

### Assessment of Reasons for Failure to Submit HRQL Assessments

In order to improve the response rate for the HRQL study, a central COG study coordinator contacted sites with enrolled patients with delinquent HRQL data. The coordinator encouraged submission of delinquent data and requested reasons for the delinquency. A second email was sent in the case of continued delinquency within the next month. A query was also generated in the case of incomplete HRQL forms.

There were two sources of comments related to non-completion of HRQL data. First, any comments that were communicated to the COG coordinator during the queries related to delinquent data were included. Second, some site CRAs provided comments directly into the COG database about why some time points were not conducted.

### Analysis

Three authors (DJ, RN and LS) independently coded all comments received. Themes were identified using thematic analysis using the methodology described by Braun and Clark [17]. The same authors also elucidated sub-themes within each theme [17], [18]. The study team met repeatedly to redefine themes and sub-themes in an iterative, continuous process. Sample quotes were identified to support themes and sub-themes.

## Results

As of January 25, 2013, there were 253 patients enrolled on AAML1031 who were eligible for the HRQL aspect of the study. Of these 253 patients, 196 consented to this aspect of the study (77.5% participation rate). The median age of these patients was 12.9 years (range 2.0 to 18.9) and 101 (51.5%) were boys. Of these 196 patients, 77 were removed from the HRQL study for the following reasons: withdrew (n = 15), deceased (n = 13), relapsed (n = 13), removed from protocol therapy for other reasons (n = 35), and site did not have the ability to gather the HRQL data (n = 1). Given the activation date of AAML1031, the number of individual HRQL assessments that were completed and the number that should have been completed at each time point from parents and patients combined were as follows: 1 (n = 959/1133); 2 (n = 749/923); 3 (n = 624/741); 4 (n = 454/578); 5 (n = 273/304); 6 (n = 64/85); 7 (n = 0); and 8 (n = 0). Of these assessments, 3123 were completed from a total of 3764 expected, giving a completion rate of 83%. Of note, no patients had reached time points 7 or 8 at the time of data collection for this analysis.

**Figure 1:**
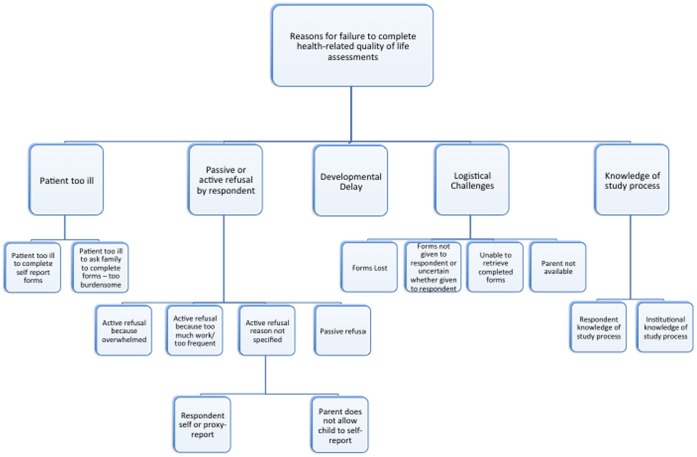
Reasons for HRQL Non-Completion. Diagram of the reasons for non-completion of health-related quality of life assessment completion.

There were sixty-two comments received that described reasons for non-completion. The reasons for non-completion were grouped into five themes (Figure 1). The five themes were: patient too ill; passive or active refusal by respondent; developmental delay; logistical challenges; and knowledge of study processes. Some comments were categorized into more than one theme. The number of comments in each theme and subtheme, as well as sample comments, is shown in Table 1.

**Table 1:** Reasons for Non-completion of Health Related Quality of Life Assessments and Sample Quotations.

Themes and Subthemes	No*	Sample Quotations
**1. PATIENT TOO ILL**		
a. Patient too ill to complete self-report forms	13	“Patient has been extremely ill in the PICU, and although approached… on three occasions, she has not been able to complete it.”
b. Patient too ill to ask family to complete forms	3	“.. he was intubated for the majority of the first time point and hospital staff did not feel it was appropriate to give these forms to his family due to their great distress…as well as the patient was unable to complete the forms himself.”
**2. REFUSAL BY RESPONDENT**		
a. Active refusal because overwhelmed	5	“Parents indicated that it was just too much for them to do.”
b. Active refusal because too much work/too frequent	2	“The main issue I have had collecting the questionnaires is the frequency.”
c. Active refusal reason not specified		
i. Respondent self or proxy report	6	“.. the patient was an active refusal (asked several times and declined to complete the questionnaires).”
ii. Parent does not allow child to self report	2	“.. the patients father stated that he did not want the child to do the survey, only the parents to complete…”
d. Passive refusal	9	“Parent approached twice but kept deferring.”
**3. DEVELOPMENTAL DELAY**	2	“.. the patients Is severely developmentally delayed to less than 2 years old and nonverbal.”
**4. LOGISTICAL CHALLENGES**		
a. Forms lost	6	“.. I think they must have took the questionnaires home or misplaced them…”
b. Forms not given to respondent or uncertainwhether given to respondent	5	“Patient discharged from hospital and window for assessment missed.”
c. Unable to retrieve completed forms	1	“…he came down with a case of C difficile colitis while the forms were in his room to fill out and now they have been put into quarantine…”
d. Parent not available	4	“…parent is not here often…’
**5. KNOWLEDGE OF STUDY PROCESS**		
a. Respondent knowledge of study process	4	“.. the patient did not fill out the form because they thought it was an extra form so they did not complete it…’
b. Institutional knowledge of study process	8	“Site misunderstood the word proxy. Thought if patient can do It, parent doesn’t need to.”

* Number of comments received.

### Patient too ill

The theme that was most frequent was that the patient was too ill to complete the assessment. This was further subcategorized into patient too ill to complete self-report forms. The majority of comments described that the patient was in the intensive care unit and often was intubated and ventilated.

*“Patient has been extremely ill in the PICU, and although approached… on three occasions, she has not been able to complete it.”*

Another subtheme in this category was that the patient was too ill to ask the family to complete the forms. It was felt that it would be too burdensome to the family to complete the HRQL forms.

*“.. he was intubated for the majority of the first time point and hospital staff did not feel it was appropriate to give these forms to his family due to their great distress….”*

### Passive or Active Refusal by Respondent

Another common theme was that of passive or active refusal by the respondent. Active refusal was subcategorized into 4 subthemes. First was active refusal because the family was overwhelmed.

*“Parents indicated that it was just too much for them to do.”*

There was also active refusal because the respondent indicated that completion of the HRQL forms was too burdensome.

*“We have two patients on this study so far and the frequency of the time points is pretty difficult for the first family.”*

In some cases the reason for the active refusal was not specified. Sometimes, a parent would not permit the child complete the HRQL assessment.

### Developmental Delay

For children with severe developmental delay, self-assessment was not possible.

### Logistical Challenges

Logistical challenges in completing the assessments were frequently described. There were four subthemes within this classification that emerged. First, the forms were lost on several occasions. A second logistical challenge encountered was that the forms were either not given to the respondent or it was uncertain whether they were given to the respondent. Third, the completed forms were unable to be retrieved from the patient and family. The fourth challenge was that the parents were rarely present in the hospital and thus, the institutional CRA was not able to provide the HRQL forms to them.

*“Patient is inpatient parent is not here often. Left HRQOL for parent to fill out but did not get returned.”*

### Knowledge of Study Process

Lack of knowledge of the study process was another important reason for non-completion of assessments. First, lack of knowledge was on the part of the respondent.

*“The patient did not fill out the form because they thought it was an extra form so they did not complete it. I think they got it confused with the parent fatigue form.”*

Second, institutional lack of knowledge of the study process was another reason for non-completion of assessments.

*“Site misunderstood the word "proxy." Thought if patient can do it, parent doesn’t need to.”*

Table 2 illustrates our recommendations for how to address the identified issues in future HRQL studies of similar patients.

**Table 2:** Suggestions for Improving Completion of HRQL Assessments.

Theme	Recommendations
Patient too ill	• Increase time frame for completion of forms
	• Provide guidance for CRAs on how to approach parents of critically ill children
	• Decrease number of assessments
	• Decrease assessment length
	• Consider CRA administration of forms
Passive or active refusal by respondent	• Increase time frame for completion of forms
	• Provide guidance for CRAs on how to approach parents of critically ill children
	• Decrease number of assessments
	• Decrease assessment length
	• Consider CRA administration of forms
Developmental delay	• Provide specific guidance on completion of self-report for children with development delay
Logistical challenges	• Use electronic patient-reported outcomes
	• Provide access for proxy reporting from remote sites
Knowledge of study processes	• Use electronic patient-reported outcomes
	• Improve educational materials

Abbreviation: CRA – clinical research associate.

## Discussion

We identified reasons for non-completion of HRQL assessments within the current COG *de novo* AML study. Our observations are relevant for at least two reasons. First, we were able to summarize reasons that HRQL assessments were not completed for an intensively treated group of children and subsequently, can provide guidance to others for how to better design similar studies. Second, we have illustrated how this methodology can efficiently identify such problems.

The most frequent reason for non-completion of HRQL assessments was that the patient was too ill to complete the evaluation or to ask the parent to provide evaluations. A potential strategy to overcome this problem is to increase the time frame during which the respondent can complete the assessment. This approach would provide more opportunities for the respondent to complete the assessment during the window for completion. Second, decreasing the number of assessments and thus minimizing respondent burden for each assessment may be helpful. Third, providing guidance and a formalized “script” to CRAs on how to approach parents of critically ill children also may be beneficial. Finally, allowing for CRA administration (where the CRA reads the questions out loud and marks off the appropriate response) may allow these evaluations to be completed.

The second most common reason for non-completion of the HRQL assessments was active or passive refusal by the patient or parent. All of the previously described strategies to address the patient being too ill may also be effective at reducing the active and passive refusal rate.

Electronic patient reported outcome (ePRO) systems use electronic data capture methods to assess topics patients can report about themselves and these may be an extremely effective approach to addressing many reasons for non-completion of HRQL assessments [19]. ePROs may lead to less administrative burden, high patient acceptance, avoidance of secondary data entry errors, easier implementation of skip patterns, electronic scoring and more accurate and complete data [19]. Study patients/families can be automatically emailed the questionnaire at the time of each scheduled assessment, ensuring timely presentation of study assessments [20]. As well, the PRO data is captured in the data file as the patient is responding to the questionnaire and any changes to the protocol can be easily accommodated in the ePROs [19].

Many logistical challenges can be overcome with ePROs, but another strategy to improve HRQL response rates is to have dedicated research staff. This has been evaluated in a previous analysis of non-therapeutic studies in cooperative group trials where funding for dedicated research staff was found to overcome many logistical challenges [21]. The benefits of dedicated research staff include optimizing response rates on HRQL studies, ensuring that the HRQL studies are a priority, and improving knowledge of the study processes.

The strength of our study is the inclusion of comments from a large number of institutions from multiple CRAs. This approach provided more generalizable data. A limitation of our study is the reliance on the institutional CRA to provide comments for reasons of non-completion. Consequently, we may have missed some important reasons for non-completion if some CRAs did not wish to provide their comments. Also, the study was only open for patients between the ages of 2 and 18, and thus, we could not evaluate reasons for non-completion of HRQL assessments specific to infants or older adolescents. Another limitation of our study is that patients with relapsed or refractory disease were removed from AAML1031 protocol therapy and were therefore not eligible to continue with the HRQL study. These patients may have particularly poor compliance with completion of HRQL assessments related to severity of illness and future research should focus on this subgroup.

In conclusion, we have described reasons for non-completion of HRQL assessments on an AML therapeutic trial in progress and provided suggestions to improve future studies. We also demonstrated that the collection of qualitative comments on reasons for non-completion of HRQL assessments is feasible.

## Supporting Information

Table S1 **List of Institutions with AAML1031 Open at Their Institution.**
(DOC)
