# Supplementary material for: Reasons for Non-Completion of Health Related Quality of Life Evaluations in Pediatric Acute Myeloid Leukemia: A Report from the Children’s Oncology Group
Source: PLoS One. 2013 Sep 6;8(9):e74549. doi: 10.1371/journal.pone.0074549 (PMC3765362; doi:10.1371/journal.pone.0074549)
Supplement: Table S1 — List of Institutions with AAML1031 Open at Their Institution. (DOC) [file pone.0074549.s001.doc]

S1 - Supplemental Table 1 - List of Centers with AAML1031 REB Approvals

Childrens Hospital and Medical Center

Albany Medical Center

Dell Children's Medical Center

Sinai Hospital - Baltimore

Saint Barnabas Medical Center

Cedars-Sinai Medical Center

Childrens Hospital of L.A.

Childrens Hospital of Orange County

Cincinnati Children's Hospital

Children's Hospital at Cleveland

City of Hope National Medical Center

Nationwide Children's Hospital

Childrens Hospital Denver

Children's Medical Center Dayton

Children's Hospital of Central California

Geisinger Medical Center

Helen DeVos Children's Hospital

Miller Children's Hospital

Penn State Hershey Children's

Riley Hospital for Children

University of Iowa Hospital

Janeway Child Health Center

Bronson Methodist Hospital

Childrens Mercy Cancer Center

Childrens Hospital of Kings Daughters

East Tennessee Childrens

Kosair Childrens Hospital

University of Kentucky

Loma Linda University Medical

Loyola University Medical Center

Children's Hem/Onc Team

Lutheran General Child Medical Center

Mary Bridge Childrens Hospital

Mayo Clinic

A.I. duPont Hospital for Children

MCG Childrens Medical Center

MeritCare Medical Group

C.S. Mott Childrens Hospital

University of Minnesota Cancer Center

Backus Children's Hospital

CancerCare Manitoba

Montefiore Medical Center

Childrens Hospital & Clinics of Mi

Mountain States Tumor Institute

Michigan State University

Vanderbilt Children's Hospital

Kaiser Foundation Research Institute

Univiversity of Nebraska Medical Center

IWK Health Centre

Toledo Children's Hospital

Newark Beth Israel Medical Center

New York University Medical Center

Children's Hospital and Research

Childrens Memorial Hospital Omaha

Atlantic Health System

Childrens Hospital of Philadelphia

Phoenix Childrens Hospital

Children's Hospital of Pittsburgh

Doernbecher Childrens Hospital

Princess Margaret Hospital

Raymond Blank Children's Hospital

South Carolina Cancer Center

Rainbow Babies Hospital

Methodist Children's Hospital

Southern California Permanente

Sydney Children's Hospital

Seattle Children's

Southern Illinois University School of Medicine

Starship Children's Hospital

St. Joseph's Hospital & Medical Center

Nevada Cancer Research Foundation CCOP

St. Vincent Children's Indiana

Mercy Children's Hospital

Connecticut Children's Medical

University of Illinois

University of North Carolina

University of California San Francisco

Primary Childrens Medical Center

New York Medical College

University of Wisconsin-AFCH

Childrens Hospital New Orleans/LSU CCO

Midwest Children's Cancer Center

Oklahoma University

B.C.'s Children's Hospital

Baptist Children's Hospital

Centre Hospitalier Universitaire

McMaster Children's Hospital

Ochsner Clinic

Tampa Children's Hospital

San Jorge Children's Hospital

The Children's Hospital Westmead

Children's Healthcare of Atlanta

Carilion Clinic Childrens Hospital

Royal Children's Hospital Brisbane

Lee Memorial Health System

Children’s Hospital of Eastern Ontario

Cook Children's Medical Center

Advocate Hope Children's Hospital

Duke University

Dartmouth Hitchcock Medical Center

Inova Fairfax Hospital

Baylor College of Medicine

Boston Floating Hospital

All Children's Hospital

Children’s Memorial Hospital (Chicago)

Wake Forest University School of Medicine

Carolinas Medical Center/Levin

Childrens Hospiotal Greenville System

Hackensack Medical Center

Johns Hopkins University

McGill University Health Center

Maine Children's Cancer Program

Joe DiMaggio Children's

Mount Sinai Medical School (N.Y.)

M. U. S. Carolina

Nemours/Jacksonville

Uiversity of New Mexico

Rhode Island Hospital

Nemours/Orlando

Roswell Park Cancer Institute

Presbyterian Hospital

Nemours Children's Clinic Pen

Scott & White Memorial Hospital

St John Hospital

SUNY Upstate Medical University

St. Vincent Hospital - Wisconsin

UT Southwestern Medical Center

UT/San Antonio

University of Alabama at Birmingham

University of Arkansas

University of California Davis

University of Florida

University of Maryland at Baltimore

University of Miami

University of Mississippi Medical Center

Eastern Maine Medical Center

University of Rochester

University of South Alabama

University of Vermont

Washington University

WV University, Charleston

Yale University

Hospital for Sick Children

Centre Hospitalier de Quebec

Schneider Children's Hospital

Florida Hospital Cancer Institute

Driscoll Children's Hospital

Emanuel Hospital

Tulane University Medical Center

Mission Hospitals

Medical City Children's Hospital

Royal Children's Hospital

University of Hawaii/Kapiolani Medical Center

Commonwealth University - MCV

Sanford Children’s Specialty

UMDNJ-Robert Wood Johnson University

Columbia University Medical Center

Broward Health Medical Center

Kingston General Hospital

Winthrop University Hospital

T.C. Thompson Children's Hospital

Sacred Heart Children's Hospital

South Island Child Cancer Serv

Saint Peter's University Hospital

M.D. Anderson Cancer Orlando

Lehigh Valley Hospital

Rocky Mountain Hospital

Mercy Hospital Saint Louis
